# Modelo de Asignación Diagnóstica y Terapéutica (MADiT; diagnostic and therapeutic assignment model): Clinical classification and 1-year mortality among older adults admitted to an acute geriatric unit: A prospective cohort study

**DOI:** 10.1016/j.jarlif.2026.100086

**Published:** 2026-09-26

**Authors:** Christian Alvarado, Enric Duaso, Isabel Prado, Andrés Gamboa, Jordi Amblas

**Affiliations:** a Geriatric Department, Hospital Universitari d’Igualada, Igualada, Spain; b Universitat de Vic – Universitat Central de Catalunya (UVic-UCC), Vic, Spain

**Keywords:** Frailty, Mortality, Geriatrics, Multimorbidity, Risk stratification, Hospitalization

## Abstract

**Background:**

Older adults admitted to acute geriatric units frequently have multidimensional geriatric vulnerability, including frailty, functional and cognitive impairment, multimorbidity, and nutritional risk. The Modelo de Asignación Diagnóstica y Terapéutica (MADiT; Diagnostic and Therapeutic Assignment Model) is a pragmatic clinical-care classification framework used in routine practice to organise pre-admission multidimensional vulnerability into four ordered categories.

**Objectives:**

To examine the association between MADiT and 1-year all-cause mortality in older adults admitted to an acute geriatric unit.

**Design:**

Single-centre prospective observational cohort study.

**Setting and participants:**

Consecutive patients aged 75 years or older admitted for acute medical illness to an acute geriatric unit between January 1, 2023, and December 31, 2024. One index admission per patient was analysed.

**Measurements:**

MADiT classification was assigned at admission according to pre-admission functional, cognitive, and clinical status and categorized as green, yellow, orange, or red. The primary outcome was 1-year all-cause mortality from the index admission. Kaplan–Meier methods and Cox proportional hazards models were used.

**Results:**

The final cohort included 430 patients, with a mean age of 87.9 ± 6.8 years; 59.4% were women. One-year mortality was 33.3% and increased across MADiT categories: 17.2% in green, 25.2% in yellow, 36.9% in orange, and 43.3% in red. Compared with the green category, the orange (adjusted HR 2.63; 95% CI 1.06–6.56) and red (adjusted HR 3.40; 95% CI 1.33–8.68) categories were associated with higher 1-year mortality after adjustment for age and sex. Adding MADiT to a prespecified base model including age, sex, and the Charlson Comorbidity Index improved model fit (likelihood-ratio χ²=11.96; df=3; p = 0.0075), reduced the Akaike information criterion from 1508.06 to 1502.10, and increased the C-statistic from 0.560 to 0.605. Confidence intervals for the C-statistics and formal calibration measures were not estimated. No statistically significant associations were observed for readmission or in-hospital complications.

**Conclusions:**

In very old patients admitted to an acute geriatric unit, baseline MADiT category was associated with a graded increase in 1-year mortality. These findings support its potential use as a pragmatic descriptive framework for baseline multidimensional geriatric vulnerability in this setting, but do not establish full psychometric validity or clinical utility. External validation is warranted.

## Highlights

• MADiT showed a graded association with 1-year mortality in geriatric inpatients.
• Orange and red categories identified patients with poorer survival.
• MADiT improved discrimination beyond a clinical base model.

## 1 Introduction

Population ageing has led to a sustained increase in the number of older adults with multimorbidity, frailty and functional dependency requiring acute hospital care [1,2]. In this population, the coexistence of chronic diseases, impaired functional reserve and biological vulnerability is associated with a higher risk of in-hospital complications, institutionalisation, readmission and mortality, particularly among patients admitted to acute geriatric units, where clinical complexity and prognostic heterogeneity are especially high [3]. Across European health systems, multimorbidity in older adults has also been consistently associated with greater healthcare utilisation, poorer self-perceived health and functional limitation [2,4].

Frailty is highly prevalent among hospitalised geriatric patients. A recent systematic review estimated that almost half of geriatric inpatients are frail, with an additional substantial proportion classified as prefrail, although prevalence varies according to care setting, patient case-mix and burden of comorbidity [3]. The combination of frailty, multimorbidity, cognitive impairment, disability and malnutrition creates a multidimensional vulnerability profile that challenges conventional disease-centred models of acute hospital care. In acute geriatric units, robust clinical stratification is therefore essential to prioritise comprehensive geriatric assessment, support discharge planning and align diagnostic and therapeutic intensity with the patient’s baseline status, functional reserve and goals of care.

Several instruments have been developed to estimate prognosis in older hospitalised patients. The Hospital Frailty Risk Score, based on routinely collected administrative data, identifies older patients at increased risk of adverse outcomes in acute care settings, whereas the Multidimensional Prognostic Index, derived from comprehensive geriatric assessment, has shown prognostic value for mortality and other clinically relevant outcomes in hospitalised older adults [5,6]. Classical prognostic indices, such as those proposed by Walter et al. and Levine et al., also estimate 1-year mortality after hospitalisation using combinations of clinical, functional and administrative variables available during or after the index admission [7,8]. However, many prognostic tools do not explicitly incorporate the diagnostic and therapeutic complexity of the acute episode or the need to tailor the intensity of care to baseline vulnerability.

This limitation is relevant because multimorbidity and complexity are not interchangeable concepts. A recent scoping review on multimorbidity in older adults accessing hospital care highlighted substantial heterogeneity in how multimorbidity is defined and measured, with complexity being explicitly addressed in only a minority of studies and most approaches relying on weighted comorbidity indices such as the Charlson Comorbidity Index [9]. In addition, nutritional vulnerability represents a major prognostic domain in hospitalised older people. The ESPEN guideline on clinical nutrition and hydration in geriatrics emphasises that malnutrition and nutritional risk are key determinants of adverse outcomes and should be systematically screened and addressed during hospitalisation [10].

The Modelo de Asignación Diagnóstica y Terapéutica (MADiT; Diagnostic and Therapeutic Assignment Model) has been proposed as a clinical-care stratification framework designed to integrate frailty, functional reserve, cognition, chronic disease burden, dependency, and selected markers of advanced illness into four ordered categories of increasing multidimensional vulnerability and clinical-care complexity [11]. Unlike approaches focused primarily on the severity of the acute disease process, MADiT is used in our Acute Geriatric Unit as a pragmatic framework that organises information from the pre-admission comprehensive geriatric assessment. Its assignment is supported, among other elements, by Frail-VIG Index thresholds [12].

MADiT classifies patients into four colour-coded categories—green, yellow, orange, and red. Operationally, green denotes preserved functional reserve and lower vulnerability (Frail-VIG <0.20); yellow denotes intermediate vulnerability (0.20 to <0.40); orange denotes marked frailty and reduced reserve (0.40 to 0.56); and red denotes severe frailty or very limited reserve (>0.56) [11,12]. These thresholds support, but do not replace, the multidimensional clinical criteria used for assignment. The framework is intended to support proportionate diagnostic and therapeutic decision-making and to avoid both age-based undertreatment and disproportionate intervention.

Despite its potential clinical usefulness, the prognostic behaviour, reproducibility, and incremental performance of the complete MADiT classification have not been fully established in acute geriatric inpatients. It also remains uncertain whether the colour categories function as reproducible staging-adjacent clinical states, rather than solely as risk tiers, and how consistently they are linked to specific care pathways in routine practice.

The objective of this study was to examine the association between MADiT clinical classification at admission and 1-year all-cause mortality in patients aged 75 years or older admitted to an acute geriatric unit. Secondary objectives were to describe its association with in-hospital mortality, in-hospital complications, and 1-year readmission. We hypothesized that higher MADiT categories, reflecting greater baseline multidimensional geriatric vulnerability, would be associated with poorer 1-year survival.

## 2 Methods

### 2.1 Study design and setting

We conducted a single-centre prospective observational cohort study in the Acute Geriatric Unit of a secondary-level university hospital. Consecutive patients admitted between January 1, 2023, and December 31, 2024, were screened for eligibility. Follow-up for the primary outcome extended to 365 days from the date of the index admission.

In routine clinical practice, MADiT was systematically applied at admission as a clinical-care stratification tool. It was used to structure the assessment and communication of pre-admission multidimensional geriatric vulnerability, functional reserve, and goals of care. MADiT was not assigned as an experimental intervention by the investigators but was embedded in usual care. Accordingly, this study examined the association between baseline MADiT classification, as used in routine clinical decision-making, and subsequent clinical outcomes.

Because MADiT is intended to characterise the patient’s baseline clinical condition before the acute episode, classification was based on pre-admission functional, cognitive and clinical status rather than solely on the immediate severity of the acute illness leading to hospitalisation.

### 2.2 Participants

All consecutive patients aged 75 years or older admitted to the Acute Geriatric Unit for acute medical illness during the study period were eligible. Consecutive inclusion was used to reflect the usual case-mix of the unit and to minimise selection bias.

To ensure independence between observations, only one index episode per patient was included. The index admission was defined as the first admission recorded during the study period and identified through the patient’s medical record number. Subsequent admissions of the same patient were not included in the primary analysis.

Admissions for surgical conditions were excluded, as were episodes in which MADiT classification could not be established because the minimum information required for its determination was unavailable. Since the aim of the study was to examine the association between a clinical-care stratification tool embedded in routine practice and subsequent outcomes, availability of a valid MADiT classification at the index admission was considered an essential methodological requirement for inclusion in the analytical cohort.

### 2.3 Variables and data sources

Data were obtained from clinical and administrative records related to the index admission. For each patient, sociodemographic variables were collected, including age, sex and place of residence before admission. Baseline functional status was assessed using the Barthel Index [13] and the Lawton–Brody Instrumental Activities of Daily Living Scale [14]. Cognitive status was assessed using the Global Deterioration Scale of Reisberg [15]. Comorbidity was described using individual comorbid conditions and the Charlson Comorbidity Index. Frailty was assessed using the Frail-VIG Index, a comprehensive geriatric assessment-based frailty instrument [12].

Baseline nutritional status was approximated using the variable previous malnutrition, defined as unintentional weight loss of at least 5% during the previous 6 months or at least 10% over a period longer than 6 months, according to information available in the clinical record. This definition was aligned with standard clinical criteria for clinically relevant weight loss in older adults and with the prognostic importance of nutritional risk in geriatric inpatients [10].

### 2.4 Main exposure: MADiT classification

The exposure of interest was MADiT classification, used in the Acute Geriatric Unit as a clinical-care stratification framework integrated into routine practice. For the primary analyses, MADiT was modelled as a four-level categorical exposure; the green category was the reference, and no linear trend across the colour categories was assumed.

MADiT classification was established at admission using information on the patient’s pre-admission baseline status, including functional capacity, cognitive status, frailty-related deficits, chronic disease burden, dependency, and selected indicators of advanced illness. The classification was intended to characterize the patient’s baseline reserve before the acute episode rather than the severity of the acute illness alone.

Patients were classified into four ordered categories: green, yellow, orange, and red. These categories represent increasing levels of multidimensional geriatric vulnerability and clinical-care complexity. Assignment was supported by structured geriatric assessment and Frail-VIG Index thresholds: green, <0.20 (preserved reserve/lower vulnerability); yellow, 0.20 to <0.40 (intermediate vulnerability); orange, 0.40 to 0.56 (marked frailty and reduced reserve); and red, >0.56 (severe frailty or very limited reserve) [11,12]. Thresholds were considered alongside the full multidimensional clinical profile.

Because Frail-VIG and comprehensive geriatric assessment components contribute to MADiT assignment, associations between MADiT categories and frailty, function, cognition, comorbidity, or nutritional vulnerability were interpreted as expected descriptive gradients within the underlying construct. They were not considered independent validation of MADiT or evidence that MADiT measures a construct separate from established geriatric vulnerability domains.

Within the intermediate yellow and orange categories, A/B profiles were defined according to prespecified clinical criteria. Profile A included patients without criteria of advanced chronic disease in the comorbidity domain of the Frail-VIG Index and/or with cognitive impairment below Global Deterioration Scale stage 5. Profile B included patients with Global Deterioration Scale stage 5 or higher and/or advanced chronic disease in the comorbidity domain of the Frail-VIG Index, or high functional dependency, defined as 4 or more points in the functional domain of the Frail-VIG Index.

MADiT assignment was recorded at admission as part of a structured comprehensive geriatric assessment. The component domains were obtained using established instruments and their conventional scoring rules, including the Barthel Index, Lawton–Brody Scale, Global Deterioration Scale, Charlson Comorbidity Index, and Frail-VIG Index. The study did not include independent duplicate MADiT ratings; therefore, inter-rater reliability of the complete classification could not be estimated in this cohort.

Operationally, MADiT functioned as decision support rather than as a mandatory treatment algorithm. Lower-complexity categories generally supported standard diagnostic evaluation, disease-directed treatment, and rehabilitation when clinically appropriate, whereas higher-complexity categories prompted more explicit review of proportionality, monitoring burden, symptom control, goals of care, and coordination with intermediate, palliative, and community services. Final decisions remained individualised. Because these care pathways were not prospectively recorded, systematic differences in care delivery across MADiT categories could not be quantified.

### 2.5 Outcomes

The primary outcome was 1-year all-cause mortality, defined as death occurring within 365 days after the index admission. For each patient, time was calculated in days from the date of index admission to death or to the end of follow-up among survivors. This outcome was selected because of its clinical relevance in very old hospitalised patients and its ability to reflect medium-term prognosis after an acute geriatric admission.

Secondary outcomes were in-hospital mortality, occurrence of at least one in-hospital complication and occurrence of at least one readmission during the year after discharge. Readmission was defined as any new hospital admission occurring within 365 days after discharge from the index episode. Because death may preclude subsequent observation of readmission, this outcome was considered susceptible to competing risk.

In-hospital complications were defined as the occurrence of at least one of the following events during admission: respiratory, infectious, cardiac, urological, gastrointestinal, electrolyte or metabolic complications, pressure ulcers or delirium. A composite variable was also created and defined as the presence of one or more complications of any type during hospitalisation.

### 2.6 Main diagnosis at admission

The main diagnosis leading to the index admission was grouped into seven mutually exclusive clinical categories to characterise the cohort and explore potential heterogeneity of outcomes according to the principal reason for hospitalisation.

Respiratory causes included infectious pneumonia, exacerbation of chronic obstructive pulmonary disease and other chronic respiratory exacerbations.

Acute cardiovascular causes included acute coronary syndrome, decompensated or new-onset heart failure and acute arrhythmias.

Non-respiratory infectious causes included urinary tract infection, sepsis, skin and soft tissue infections and other non-respiratory infections.

Geriatric syndromes included acute functional decline, recurrent falls, immobility and delirium.

Neurological or cerebrovascular causes included ischaemic or haemorrhagic stroke, transient ischaemic attack, seizures and encephalopathies.

Metabolic or renal causes included electrolyte disorders, dehydration, decompensated chronic kidney disease and diabetic decompensation.

Other causes included diagnoses not classifiable in the previous categories.

This grouping was used for descriptive and exploratory analytical purposes, without assuming that admission diagnosis alone explained the association between MADiT classification and clinical outcomes.

### 2.7 Statistical analysis

Continuous variables were summarised as mean and standard deviation or median and interquartile range, according to their distribution. Categorical variables were described as absolute frequencies and percentages. Baseline characteristics were presented for the overall cohort and stratified by MADiT category.

One-year survival was estimated using Kaplan–Meier curves and compared across MADiT categories with the log-rank test. The primary estimand was the category-specific relative hazard of 1-year all-cause mortality associated with baseline MADiT classification. Cox proportional hazards models included three indicator variables for the yellow, orange, and red categories, with green as the reference. Sequential models comprised an unadjusted model and a model adjusted for age and sex. The proportional hazards assumption was assessed using Schoenfeld residuals.

Because MADiT integrates domains closely related to frailty, functional status, cognition and baseline clinical reserve, the primary adjusted analysis was intentionally parsimonious to avoid substantial overlap between the exposure of interest and variables contributing to its construction. The results were therefore interpreted as observed clinical associations rather than independent causal estimates.

To explore the incremental contribution of MADiT beyond a prespecified clinical base model, a Cox model including age, sex, and the Charlson Comorbidity Index was compared with an extended model that additionally included MADiT as a categorical variable. Model comparison used the likelihood-ratio test, Akaike information criterion, and the C-statistic for censored survival data. Confidence intervals for the C-statistics, formal calibration measures, and optimism-corrected performance estimates were not obtained; therefore, this analysis was considered exploratory and represents apparent performance in the derivation cohort.

In-hospital mortality, occurrence of at least one in-hospital complication and readmission during the year after discharge were analysed as secondary outcomes. The association between MADiT categories and in-hospital complications was explored by comparing proportions using Pearson’s chi-square test.

For readmission, the primary analysis used a Fine–Gray competing-risk model, considering death as a competing event and reporting subdistribution hazard ratios with 95% confidence intervals. As a sensitivity analysis, cause-specific hazard models were estimated, censoring patients at the time of death.

Analyses of A/B profiles within the intermediate MADiT categories and analyses stratified by admission diagnosis were considered exploratory. No interaction between MADiT and treatment or care pathway was estimated; consequently, the study evaluated baseline prognostic classification rather than differential treatment effects or effect modification.

All tests were two-sided, and a p value <0.05 was considered statistically significant. Participants without a recorded death by day 365 were administratively censored at 365 days. No missing-data imputation was performed; analyses used available cases, and denominators were reported when data were incomplete. Statistical analyses were performed using IBM SPSS Statistics for Windows, Version 27.0 (IBM Corp., Armonk, NY, USA; 2020) and R software (R Foundation for Statistical Computing, Vienna, Austria; 2024). Survival analyses, assessment of the proportional hazards assumption, competing-risk models, and discrimination statistics for survival data were conducted in R.

### 2.8 Sample size

Sample size was estimated for the primary outcome, 1-year all-cause mortality, using the Schoenfeld approximation for Cox proportional hazards models. Assuming an annual mortality rate of approximately 40% in the overall cohort and an estimated distribution of approximately 70% of patients in higher-complexity MADiT categories, a minimum sample size of 256 patients, with approximately 113 events, was estimated to be required to detect a hazard ratio of 1.8 between lower- and higher-complexity categories, with a two-sided alpha level of 0.05 and 80% power.

Nevertheless, given the observational nature of the study and its conduct in a real-world clinical setting, all consecutive eligible patients admitted during the study period were included. This strategy yielded a final analytical cohort of 430 patients with available MADiT classification, exceeding the minimum estimated sample size required for the primary analysis.

The sample size calculation was interpreted as an assessment of adequacy for the primary outcome and not as a guarantee of statistical power for secondary or exploratory analyses, particularly those involving small subgroups such as some colour categories and A/B profiles.

### 2.9 Ethical considerations

The study was approved by the Research Ethics Committee of Bellvitge University Hospital (PR30/2022) and was conducted in accordance with the principles of the Declaration of Helsinki and applicable data protection regulations.

Written informed consent was obtained during hospitalisation. In patients with cognitive impairment or inability to understand study participation, consent was obtained from the legal representative.

## 3 Results

### 3.1 Study population

During the study period, 927 consecutive admission episodes to the Acute Geriatric Unit were recorded. After selecting a single index admission per patient, 448 unique patients were identified. Of these, 18 patients were excluded because a valid MADiT classification could not be established owing to insufficient information at the index episode. The final analytical cohort therefore included 430 patients. The patient flow diagram is shown in Fig. 1.

**Fig. 1:**
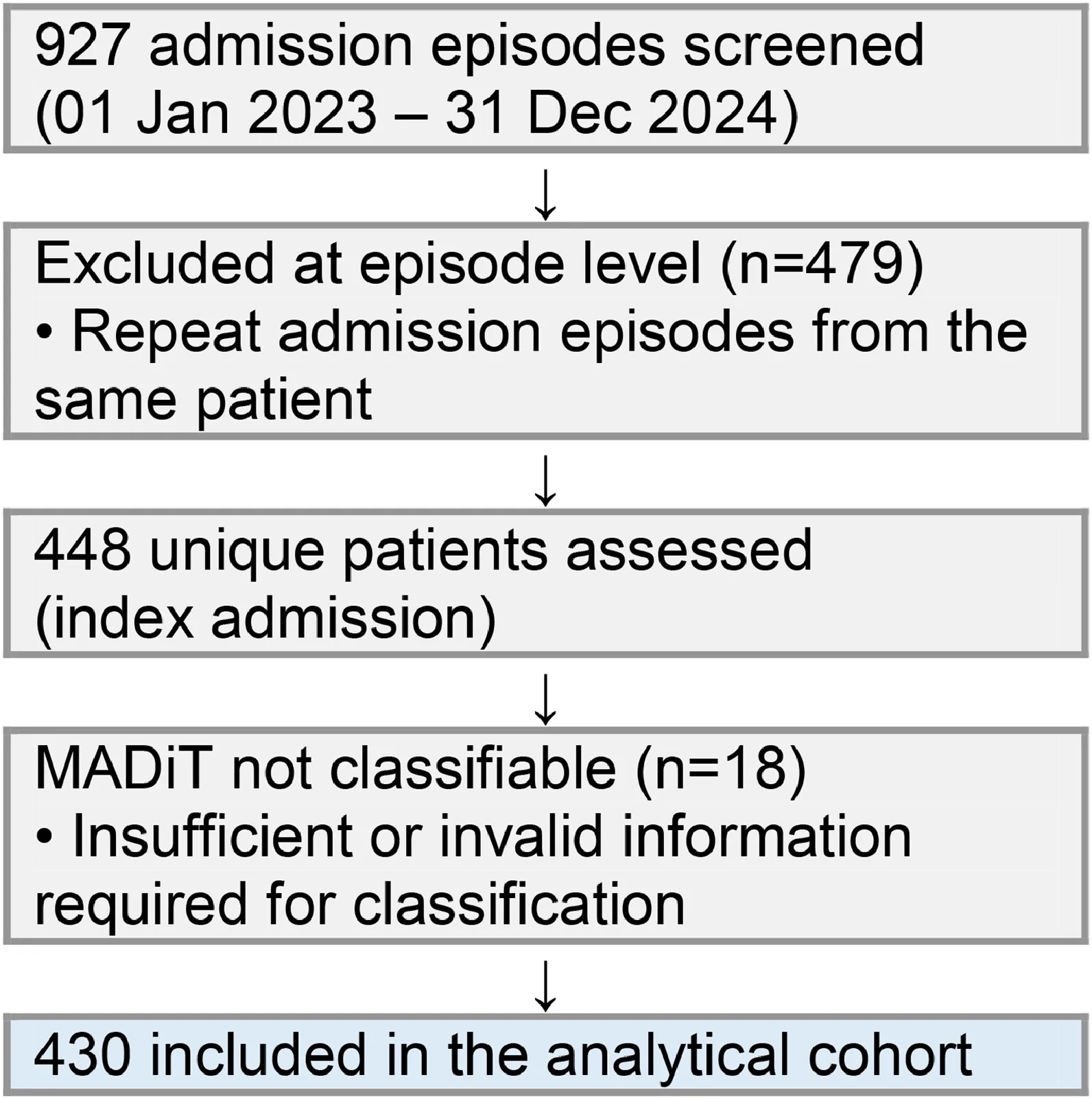
Study flow diagram.

**Abbreviations:** AGU, Acute Geriatric Unit; MADiT, Modelo de Asignación Diagnóstica y Terapéutica (Diagnostic and Therapeutic Assignment Model); Frail-VIG, Frailty Index based on Comprehensive Geriatric Assessment.

### 3.2 Baseline characteristics

The final analytical cohort included 430 patients, with a mean age of 87.9 ± 6.8 years; 255 patients were women. The distribution across MADiT categories was as follows: green, 29 patients (6.7%); yellow, 135 patients (31.4%); orange, 176 patients (40.9%); and red, 90 patients (20.9%).

There were no statistically significant differences across MADiT categories in age or sex. In contrast, higher-complexity MADiT categories showed progressively worse baseline geriatric status. Increasing MADiT complexity was associated with lower Barthel Index and Lawton–Brody scores, higher Charlson Comorbidity Index, higher Frail-VIG Index, worse cognitive status according to the Global Deterioration Scale, and a higher prevalence of previous malnutrition; all global comparisons were statistically significant. Institutional residence before admission was more frequent in the orange and red categories, whereas the green category consisted almost entirely of patients admitted from home (Table 1).

**Table 1:** Baseline characteristics of the cohort according to MADiT category.

Variable	Green (n = 29)	Yellow (n = 135)	Orange (n = 176)	Red (n = 90)	p value
Age, years	88.62 ± 3.49	89.71 ± 5.38	88.55 ± 6.43	86.28 ± 8.95	0.079
Female sex	12 (41.4%)	77 (57.0%)	115 (65.3%)	56 (62.2%)	0.070
Baseline Barthel Index	94.83 ± 6.05	77.89 ± 20.59	46.56 ± 18.89	27.91 ± 22.01	<0.001
Lawton–Brody Index	5.32 ± 1.72	2.55 ± 1.74	1.14 ± 1.14	0.61 ± 0.90	<0.001
Charlson Comorbidity Index	6.04 ± 1.67	7.35 ± 1.63	8.05 ± 1.30	8.65 ± 1.47	<0.001
Frail-VIG Index	0.12 ± 0.05	0.31 ± 0.07	0.45 ± 0.05	0.62 ± 0.07	<0.001
Global Deterioration Scale	1.07 ± 0.70	2.39 ± 1.55	3.61 ± 1.69	4.47 ± 1.91	<0.001
Previous malnutrition	2 (6.9%)	19 (14.1%)	37/176 (21.1%)	40/90 (44.4%)	<0.001
Residence before admission: home	28 (96.6%)	112 (83.0%)	86 (48.9%)	44 (48.9%)	<0.001
Residence before admission: nursing home	1/29 (3.4%)	22/135 (16.3%)	86/176 (48.9%)	44/90 (48.9%)	<0.001
Residence before admission: intermediate care	0/29 (0.0%)	1/135 (0.7%)	4/176 (2.3%)	2/90 (2.2%)	0.609

**Abbreviations:** MADiT, Modelo de Asignación Diagnóstica y Terapéutica (Diagnostic and Therapeutic Assignment Model); Frail-VIG, Frailty Index based on Comprehensive Geriatric Assessment; SD, standard deviation.

As expected from the construction of the MADiT classification, higher categories showed progressively greater impairment across baseline geriatric vulnerability domains.

### 3.3 Admission diagnosis, length of stay and in-hospital complications

The distribution of the main diagnosis at admission differed across MADiT categories (p = 0.002) (Table 2). Acute cardiovascular conditions were the most frequent reason for admission in the green and yellow categories, whereas non-respiratory infections were relatively more frequent in the red category. Other diagnostic categories showed a more heterogeneous distribution.

**Table 2:** Admission diagnosis, length of stay and in-hospital complications according to MADiT category.

Variable	Green (n = 29)	Yellow (n = 135)	Orange (n = 176)	Red (n = 90)	p value
Main diagnosis at admission					0.002
Respiratory	12 (41.4%)	30 (22.2%)	58 (33.0%)	30 (33.3%)	0.077
Acute cardiovascular	12 (41.4%)	62 (45.9%)	60 (34.1%)	25 (27.8%)	0.032
Non-respiratory infection	0 (0.0%)	12 (8.9%)	23 (13.1%)	21 (23.3%)	0.002
Geriatric syndromes	2 (6.9%)	18 (13.3%)	15 (8.5%)	3 (3.3%)	0.074
Neurological/cerebrovascular	3 (10.3%)	7 (5.2%)	6 (3.4%)	3 (3.3%)	0.351
Metabolic/renal	0 (0.0%)	3 (2.2%)	12 (6.8%)	7 (7.8%)	0.100
Other	0 (0.0%)	3 (2.2%)	2 (1.1%)	1 (1.1%)	0.748
Length of stay, days, median (IQR)	8 [[3], [4], [5], [6], [7], [8], [9], [10], [11]]	6 [[4], [5], [6], [7], [8], [9]]	6 [[4], [5], [6], [7], [8]]	7 [[3], [4], [5], [6], [7], [8], [9]]	0.403
≥1 in-hospital complication	2 (6.9%)	14 (10.4%)	19 (10.8%)	8 (8.9%)	0.901

**Abbreviations:** MADiT, Modelo de Asignación Diagnóstica y Terapéutica (Diagnostic and Therapeutic Assignment Model); IQR, interquartile range.

Median length of hospital stay did not differ significantly across MADiT categories. Similarly, the proportion of patients experiencing at least one in-hospital complication was comparable across groups, with no evidence of a statistically significant global association (Table 2).

### 3.4 In-hospital mortality and 1-year mortality

In-hospital mortality increased across MADiT categories, from 3.4% in the green category to 14.4% in the red category. However, the global difference across categories did not reach statistical significance (p = 0.057).

During the 365-day follow-up period, 143 of 430 patients died, corresponding to an overall 1-year all-cause mortality of 33.3%. One-year mortality increased progressively across MADiT categories: 17.2% in green, 25.2% in yellow, 36.9% in orange and 43.3% in red (global p = 0.0058). The distribution of 1-year mortality across MADiT categories is shown in Fig. 2.

**Fig. 2:**
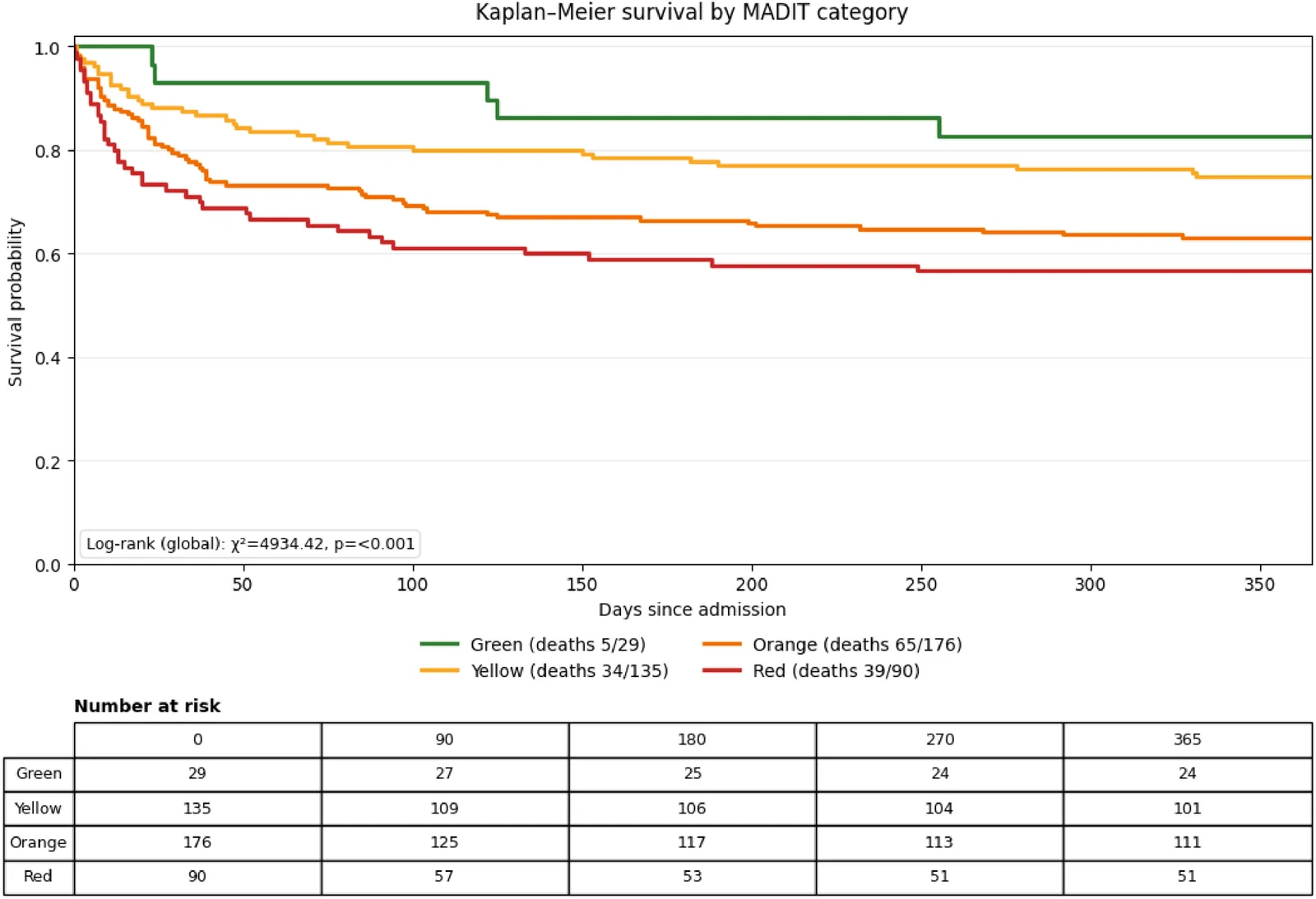
Kaplan–Meier survival curves according to baseline MADiT category.

MADiT categories represent increasing baseline multidimensional vulnerability and clinical-care complexity: green, Frail-VIG <0.20 (preserved reserve/lower vulnerability); yellow, 0.20 to <0.40 (intermediate vulnerability); orange, 0.40 to 0.56 (marked frailty and reduced reserve); and red, >0.56 (severe frailty or very limited reserve). Thresholds supported assignment but did not replace the full multidimensional criteria; see Methods. Abbreviation: MADiT, Modelo de Asignación Diagnóstica y Terapéutica (Diagnostic and Therapeutic Assignment Model).

### 3.5 Association between MADiT and 1-year mortality

Kaplan–Meier curves showed progressive separation across MADiT categories, with lower 1-year survival in higher-complexity groups. The global comparison of survival curves supported differences in survival according to MADiT classification.

In the unadjusted Cox proportional hazards model, MADiT classification was globally associated with 1-year mortality (p = 0.004). Compared with the green category, the yellow category was not significantly associated with mortality (HR 1.58; 95% CI 0.62–4.04; p = 0.340). In contrast, the orange category (HR 2.52; 95% CI 1.02–6.27; p = 0.046) and the red category (HR 3.22; 95% CI 1.27–8.18; p = 0.014) were associated with higher 1-year mortality (Table 3).

**Table 3:** Association between MADiT categories and 1-year all-cause mortality: Cox proportional hazards models.

MADiT category	Unadjusted HR	95% CI	p value	Age- and sex-adjusted HR	95% CI	p value
Green	Reference	—	—	Reference	—	—
Yellow	1.58	0.62–4.04	0.340	1.60	0.62–4.11	0.329
Orange	2.52	1.02–6.27	0.046	2.63	1.06–6.56	0.038
Red	3.22	1.27–8.18	0.014	3.40	1.33–8.68	0.010

**Abbreviations:** CI, confidence interval; HR, hazard ratio; MADiT, Modelo de Asignación Diagnóstica y Terapéutica (Diagnostic and Therapeutic Assignment Model).

After adjustment for age and sex, the direction and magnitude of the associations remained similar. Compared with the green category, the yellow category was not significantly associated with 1-year mortality (HR 1.60; 95% CI 0.62–4.11; p = 0.329). In contrast, the orange category (HR 2.63; 95% CI 1.06–6.56; p = 0.038) and the red category (HR 3.40; 95% CI 1.33–8.68; p = 0.010) remained associated with higher 1-year mortality. Neither age nor sex was independently associated with mortality in this model.

To explore the incremental contribution of MADiT beyond the prespecified base model including age, sex, and the Charlson Comorbidity Index, the base model was compared with an extended model additionally including MADiT classification. Adding MADiT improved global model fit (likelihood-ratio χ²=11.96; df=3; p = 0.0075), reduced the Akaike information criterion from 1508.06 to 1502.10, and modestly increased discrimination, with the C-statistic rising from 0.560 to 0.605 (absolute change, 0.045). Confidence intervals for the C-statistics, formal calibration measures, and optimism-corrected estimates were not available. Accordingly, these figures describe apparent performance in the same cohort and should be interpreted as exploratory.

### 3.6 A/B profiles within intermediate MADiT categories

In the exploratory analysis of A/B profiles within intermediate MADiT categories, differences in 1-year mortality were observed within the yellow category. Patients classified as yellow profile B had higher 1-year mortality than those classified as yellow profile A: 38.3% (23/60) versus 15.3% (11/72), respectively (p = 0.005). This finding was consistent with the survival analysis (log-rank p = 0.007).

Within the orange category, 1-year mortality was numerically higher in profile B than in profile A: 38.6% (61/158) versus 23.5% (4/17), respectively. However, this difference was not statistically significant (Fisher’s exact test p = 0.294; log-rank p = 0.572). Interpretation of this result is limited by the marked imbalance in subgroup size (Fig. 3).

**Fig. 3:**
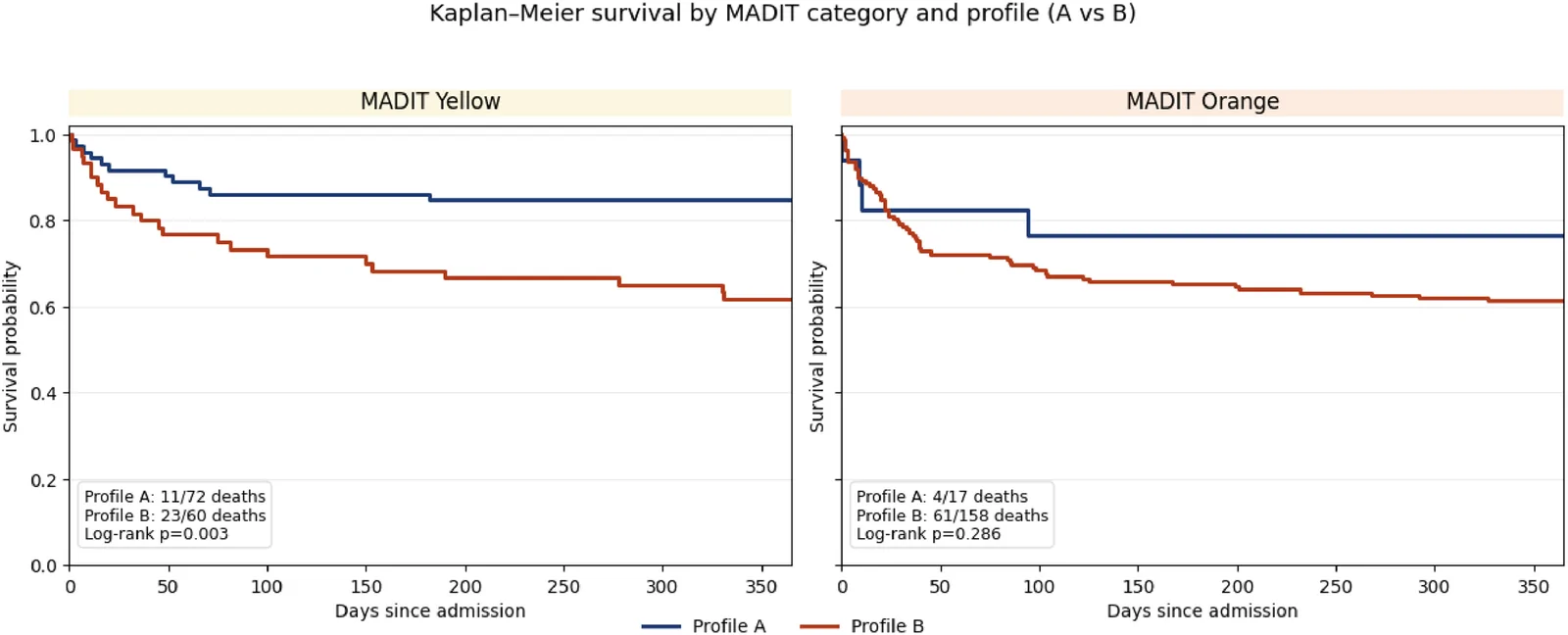
Kaplan–Meier survival according to A/B profile within the yellow and orange MADiT categories.

The yellow category corresponds to Frail-VIG 0.20 to <0.40 and the orange category to 0.40 to 0.56. Profile A included patients without advanced chronic disease criteria in the Frail-VIG comorbidity domain and/or with cognitive impairment below Global Deterioration Scale stage 5. Profile B included patients with Global Deterioration Scale stage 5 or higher and/or advanced chronic disease, or high functional dependency (≥4 points in the Frail-VIG functional domain). See Methods for the complete operational definition. Abbreviation: MADiT, Modelo de Asignación Diagnóstica y Terapéutica (Diagnostic and Therapeutic Assignment Model).

### 3.7 Exploratory analyses

No statistically significant associations were observed between MADiT categories and the occurrence of at least one readmission during the year after discharge or with the occurrence of in-hospital complications. These findings were consistent with the absence of a clear signal in the corresponding descriptive analyses.

One-year mortality stratified by admission diagnosis showed heterogeneity across diagnostic groups, with a general pattern of higher mortality in higher-complexity MADiT categories. However, differences across diagnostic subgroups did not reach statistical significance, and these analyses should be interpreted as exploratory (**Supplementary Figure 1**).

## 4 Discussion

In this single-centre prospective observational cohort of very old patients admitted to an Acute Geriatric Unit, higher MADiT clinical classification categories were associated with higher 1-year all-cause mortality. Mortality increased progressively from the green to the red category, and the orange and red categories were associated with poorer survival after adjustment for age and sex. These findings indicate that MADiT classification identifies groups with differing baseline multidimensional geriatric vulnerability and subsequent survival in this clinical setting.

The observed mortality gradient is clinically coherent but partly expected from the underlying structure of the classification. Similarly, the graded differences in Barthel Index, Lawton–Brody score, Global Deterioration Scale, Frail-VIG Index, comorbidity burden, and previous malnutrition across MADiT categories represent expected descriptive features of the framework rather than independent evidence of construct validity [[5], [6], [7], [8],^16^,^17^]. This distinction is important. Our findings support the potential clinical relevance of MADiT as a marker of vulnerability and complexity, but they should not be interpreted as evidence of an independent causal effect of the classification itself.

Conceptually, MADiT is staging-adjacent rather than a formal disease-staging system or a simple aggregate risk score. Higher categories broadly parallel increasing severity across established geriatric domains, including frailty, disability, cognitive impairment, advanced chronic disease, and dependency. However, the categories do not represent mutually exclusive syndromic stages, fixed biological progression, or an inevitable clinical trajectory. Longitudinal change in MADiT category should therefore be interpreted only after reassessment of the underlying multidimensional clinical state.

The scope of validation must also be distinguished. The mortality gradient provides evidence of prognostic criterion validity and clinical construct coherence within this cohort, but the study was not designed as a full psychometric validation of MADiT. The original MADiT publication describes the clinical rationale and four-category framework, whereas published measurement evidence relates principally to the Frail-VIG Index that contributes to assignment [11,12]. We did not evaluate factor structure, internal consistency, inter-rater or test–retest reliability, or measurement invariance of the complete MADiT classification.

These findings are consistent with previous literature showing that frailty, multimorbidity and multidimensional vulnerability are strongly associated with adverse outcomes in older hospitalised adults. The Hospital Frailty Risk Score has shown utility for identifying older patients at increased risk of adverse hospital outcomes using routinely collected data, whereas the Multidimensional Prognostic Index, derived from comprehensive geriatric assessment, has demonstrated prognostic value for mortality and other relevant outcomes in geriatric inpatients [5,6,18]. Prospective studies in acute care settings have also confirmed that frailty is associated with mortality and readmission, supporting the prognostic importance of baseline vulnerability beyond the index diagnosis [19]. Within this context, MADiT does not necessarily compete with existing prognostic instruments; rather, it may occupy a complementary space by combining baseline geriatric vulnerability with a practical framework for clinical-care stratification [11].

The exploratory analysis of A/B profiles within the intermediate MADiT categories provides an additional signal of interest. In the yellow category, profile B was associated with higher 1-year mortality than profile A, suggesting that cognitive impairment, advanced chronic disease and marked functional dependency may refine risk stratification within intermediate levels of complexity. In the orange category, mortality was numerically higher in profile B than in profile A, although the difference was not statistically significant. This lack of statistical significance should be interpreted cautiously because of the substantial imbalance in subgroup size and the exploratory nature of the analysis.

In contrast, MADiT was not significantly associated with in-hospital complications or readmission during the year after discharge. Several explanations may account for these findings. First, the overall frequency of in-hospital complications was relatively low, and the composite definition combined clinically heterogeneous events, potentially reducing sensitivity to detect differences across categories. Second, readmission is a complex outcome influenced not only by frailty and disease burden but also by social support, care transitions, access to community services and end-of-life care planning. Moreover, death is a competing event that may preclude the observation of readmission, particularly in cohorts with high mortality. The absence of a robust association with readmission is therefore not unexpected and is consistent with previous research suggesting that frailty-related tools often show stronger and more consistent associations with mortality than with unplanned readmissions [20].

The heterogeneity of 1-year mortality according to admission diagnosis suggests that MADiT may capture vulnerability dimensions that cut across acute disease categories. In other words, the risk gradient associated with higher MADiT complexity appeared to persist, with varying magnitude, across different reasons for admission. This interpretation is consistent with evidence indicating that frailty and multimorbidity increase mortality risk in hospitalised older adults, partly independently of the index diagnosis [19,21]. However, analyses stratified by admission diagnosis were exploratory and probably underpowered; therefore, these findings should be considered hypothesis-generating.

An important aspect of the present study is the graded clinical coherence observed across MADiT categories. Higher-complexity categories were associated with progressively poorer baseline functional status, greater cognitive impairment, higher comorbidity burden, greater frailty, and a higher prevalence of previous malnutrition. This pattern supports the clinical plausibility of MADiT as a composite marker of geriatric complexity, but should not be interpreted as psychometric internal consistency because several of these domains contribute directly to category assignment. The coexistence of frailty and malnutrition is particularly relevant because both domains have been associated with mortality, prolonged hospitalisation, and adverse outcomes in older inpatients [10,22,23].

The potential value of MADiT lies not only in risk ordering but also in providing a shared clinical language for proportional decision-making. In routine use, lower-complexity categories may support standard diagnostic work-up, disease-directed treatment, and rehabilitation when clinically appropriate; higher-complexity categories may prompt explicit review of monitoring burden, symptom-focused care, goals-of-care discussions, and coordination with intermediate, palliative, or community services. MADiT should not determine these decisions automatically, and treatment should remain individualised. Because the relevant care processes were not prospectively measured, this study cannot establish whether MADiT changed care delivery or improved patient outcomes. Moreover, because MADiT was embedded in routine decision-making, the association with mortality may reflect both baseline vulnerability and downstream care decisions, creating potential confounding by indication.

This study has several strengths. It included a consecutive cohort of very old patients admitted to an Acute Geriatric Unit, with prospective inclusion, systematic MADiT classification at admission and clinically meaningful 1-year follow-up. The cohort was characterised across key geriatric domains, including functional status, cognition, comorbidity, frailty and nutritional status. The use of survival analysis, competing-risk methods for readmission and assessment of incremental model performance also strengthens the methodological approach.

Several limitations should be acknowledged. First, the single-centre design and local embedding of MADiT limit external validity; apparent performance may differ across hospitals, health systems, case-mix profiles, and documentation practices. Second, the small green category reduced the precision of category-specific hazard ratios. Third, the parsimonious age- and sex-adjusted model reduced overlap with domains embedded in MADiT but did not eliminate residual confounding. Fourth, MADiT was part of routine clinical decision-making, and care pathways were not prospectively recorded; its stratifying value cannot therefore be separated from the effects of decisions influenced by the classification. Fifth, no independent duplicate MADiT ratings were obtained, precluding assessment of inter-rater reliability, and the study did not evaluate the broader psychometric properties of the complete framework. Sixth, the incremental performance analysis used the same cohort without confidence intervals for the C-statistics, formal calibration assessment, or correction for optimism. Finally, A/B profile and admission-diagnosis analyses were exploratory and require confirmation in larger samples.

In summary, baseline MADiT classification, used as a clinical-care stratification framework in an Acute Geriatric Unit, was associated with a graded increase in 1-year all-cause mortality. The study provides preliminary evidence of prognostic criterion validity in this cohort, but it does not constitute a full psychometric validation, demonstrate treatment-effect modification, or establish clinical utility. Before broader implementation as a decision-support tool, MADiT requires multicentre external validation, calibration assessment, reproducibility studies, and direct comparison with established geriatric prognostic instruments [5,6,11,18].

## 5 Conclusions

In this prospective observational cohort of older adults admitted to an Acute Geriatric Unit, baseline MADiT classification was associated with a graded increase in 1-year all-cause mortality. Higher categories identified patients with greater multidimensional geriatric vulnerability and poorer survival. Adding MADiT to a prespecified model including age, sex, and the Charlson Comorbidity Index improved model fit and increased the apparent C-statistic from 0.560 to 0.605.

These findings support MADiT as a pragmatic descriptive classification framework in this specific acute geriatric setting. They do not demonstrate that MADiT is an independent prognostic instrument, that it outperforms established geriatric assessment tools, that it modifies treatment effects, or that its use improves clinical outcomes. Multicentre external validation, calibration assessment, formal reproducibility studies, direct comparison with established prognostic instruments, and prospective evaluation of clinical utility are required before broader use as a decision-support framework can be recommended.

## Declaration of generative AI and AI-assisted technologies in the writing process

During the revision of this manuscript, the authors used a generative AI tool solely for language editing and editorial refinement. The tool was not used for study design, data collection, statistical analysis, interpretation of the results, or generation of scientific conclusions. All AI-assisted text was critically reviewed and edited by the authors, who take full responsibility for the content of the publication.

## Ethics approval

The study was approved by the Research Ethics Committee of Bellvitge University Hospital, Barcelona, Spain (approval code PR30/2022), and was conducted in accordance with the Declaration of Helsinki and applicable European and Spanish data protection regulations.

## Consent to participate

Written informed consent was obtained from all participants during hospitalisation. In patients with cognitive impairment or inability to provide informed consent, consent was obtained from the legal representative.

## Consent for publication

Not applicable. This manuscript does not contain identifiable individual patient data.

## Strobe statement

Reporting guideline

This study is reported in accordance with the Strengthening the Reporting of Observational Studies in Epidemiology (STROBE) statement for cohort studies.

## Funding

This research received no specific grant from any funding agency in the public, commercial, or not-for-profit sectors.

## CRediT authorship contribution statement

**Christian Alvarado:** Writing – original draft, Software, Methodology, Investigation, Funding acquisition, Formal analysis, Data curation, Conceptualization. **Enric Duaso:** Writing – review & editing, Validation, Supervision, Project administration, Methodology, Investigation, Conceptualization. **Isabel Prado:** Writing – review & editing, Resources. **Andrés Gamboa:** Writing – review & editing. **Jordi Amblas:** Writing – review & editing, Validation, Supervision, Project administration.

## Declaration of competing interest

The authors declare the following financial interests/personal relationships which may be considered as potential competing interests:

Christian Alvarado Escobar reports equipment, drugs, or supplies was provided by Igualada University Hospital. If there are other authors, they declare that they have no known competing financial interests or personal relationships that could have appeared to influence the work reported in this paper.

## Data Availability

The data that support the findings of this study are not publicly available because they contain clinical information from hospitalized patients and are subject to institutional, ethical, and data-protection restrictions. De-identified data may be made available by the corresponding author upon reasonable request, subject to approval by the relevant institutional and ethics authorities and in accordance with applicable data-protection regulations.
