# Supplementary material for: Modelo de Asignación Diagnóstica y Terapéutica (MADiT; diagnostic and therapeutic assignment model): Clinical classification and 1-year mortality among older adults admitted to an acute geriatric unit: A prospective cohort study
Source: JAR Life. 2026 Sep 26;15:100086. doi: 10.1016/j.jarlif.2026.100086 (PMC13635708; doi:10.1016/j.jarlif.2026.100086)
Supplement: Supplementary file 1 [file mmc1.docx]

**Supplementary Figure** **1**. One-year mortality by admission diagnosis, stratified by MADiT category

Bars represent observed 1-year mortality proportions; error bars indicate Wilson 95% confidence intervals. MADiT categories represent increasing baseline multidimensional vulnerability and clinical-care complexity: green, Frail-VIG <0.20 (preserved reserve/lower vulnerability); yellow, 0.20 to <0.40 (intermediate vulnerability); orange, 0.40 to 0.56 (marked frailty and reduced reserve); and red, >0.56 (severe frailty or very limited reserve). Thresholds supported assignment but did not replace the full multidimensional criteria; see Methods. Admission-diagnosis groups are ordered by overall mortality.
